# Higher aged neutrophils and differential inflammatory profiles in sickle cell disease patients on chronic transfusion therapy versus those on hydroxyurea

**DOI:** 10.3389/fimmu.2025.1671061

**Published:** 2025-09-26

**Authors:** Katrina Terrigno, Zachary N. Flamholz, Aakash Mahant Mahant, Ilir Agalliu, Jennifer De Los Santos, Karen Ireland, Janine Keenan, Jacob S. Kazmi, Anayeli Correa, Paul S. Frenette, Libusha Kelly, Betsy C. Herold, Deepa Manwani

**Affiliations:** ^1^ Department of Pediatrics, Children’s Hospital at Montefiore and Albert Einstein College of Medicine, Bronx, NY, United States; ^2^ Department of Systems and Computational Biology, Albert Einstein College of Medicine, Bronx, NY, United States; ^3^ Department of Microbiology and Immunology, Albert Einstein College of Medicine, Bronx, NY, United States; ^4^ Department of Epidemiology and Population Health, Albert Einstein College of Medicine, Bronx, NY, United States; ^5^ Department of Cell Biology, Albert Einstein College of Medicine, Bronx, NY, United States; ^6^ Department of Hematology, St Jude Children’s Research Hospital, Memphis, TN, United States

**Keywords:** sickle cell disease, inflammatory cytokines, aged neutrophils, hydroxyurea, chronic transfusion, inflammation, gut microbiome

## Abstract

**Background:**

Sickle cell disease (SCD) is characterized by a point mutation in the β globin molecule, causing the sickling of red blood cells, and leading to hemolytic anemia, pain, and end-organ damage. Hydroxyurea (HU) is a cornerstone of SCD patient treatment, while chronic transfusions (CT) are used as part of treatment for more severe SCD. Increases in aged neutrophils and inflammation have been linked to more severe SCD and contribute to vaso-occlusive crises. The current study was designed to test the hypothesis that HU reduces inflammation and aged neutrophils.

**Study design:**

We compared clinical characteristics, aged neutrophils, levels of select cytokines, chemokines, and cell adhesion molecules in the blood and the Shannon diversity index (SDI) and ratio of Firmicutes/Bacteroides (F:B) in stool samples from pediatric SCD patients treated with HU (n=40) versus CT (n=14).

**Results:**

Patients in the HU group had significantly lower total and aged neutrophils (*p*<0.0001) compared to the CT group and also had lower levels of several chemokines including CXCL10 (IP-10), CCL2 (MCP-1) and CCL4 (MIP-1β) as well as IFN-γ and IL10. Conversely, HU was associated with higher levels of IL-1α, IL-6 and IL-8. There were no significant differences in cell adhesion markers or in markers of gut microbial dysbiosis between treatment groups. In a multivariable linear regression model, only being on CT was associated with increased number of aged neutrophils (p<0.001) whereas being on CT and having a lower SDI were associated with higher total neutrophil count.

**Discussion:**

Lower numbers of total and aged neutrophils and lower levels of several cytokines and chemokines in the HU group highlight the drug’s potential to modulate leukocyte activation and recruitment. These findings suggest that adding or maintaining HU therapy in SCD patients undergoing CT could potentially enhance immunologic regulation and warrants further study.

## Background

Sickle cell disease (SCD) is characterized by a point mutation in the β globin molecule, leading to hemolytic anemia, pain, and end-organ damage. This condition is also associated with increased inflammation, marked by the recruitment of leukocytes, including neutrophils. These cells contribute to vaso-occlusion, which exacerbates the pain and organ damage in affected patients. Hydroxyurea (HU) has become a cornerstone of SCD treatment. It functions primarily by reactivating fetal hemoglobin expression, thereby blocking polymerization of sickle hemoglobin. HU has been shown to have additional beneficial effects by suppressing neutrophil activation and reducing expression of adhesion molecules on red blood cells and endothelial cells (1), thus reducing inflammation and further preventing vaso-occlusion. When patients develop more severe disease, such as frequent pain episodes or acute chest syndrome, despite HU therapy, or stroke, they are often transitioned from HU to chronic transfusion (CT) therapy as a strategy for intensifying disease modifying therapy. CT therapy is also used for primary stroke prophylaxis in high-risk patients based on the results of the Stroke Prevention (STOP) trial (2).

There are limited studies directly comparing the effects of HU versus (vs) CT on soluble immune markers including cytokines, chemokines, and adhesion molecules in SCD patients. Nickel et al. compared numbers of lymphocyte and leucocyte subsets in patients on HU vs CT and found lower numbers in the HU cohort (3). Dembélé et al. reported on a limited panel (TNF-α, IL-8 and IL-1β) and compared CT patients to SCD patients not on HU and saw essentially no differences. They also saw no difference in aged neutrophils (AN), the subset of neutrophils that have been shown to contribute to vaso-occlusion and inflammation, comparing different treatment cohorts (CT vs HU vs combination of CT and HU). However, they did not report on potential impact of confounding variables such as age, race, absolute neutrophil count, and iron overload (4). Few studies have investigated the potential benefits or risks of continuing HU with CT. Those that have explored this combination therapy have observed reduced blood transfusion requirements and improved markers of hemolysis (5) as well as reduced rates of venous thromboembolism in SCD patients with central venous access devices, suggesting improved endothelial health and inflammation (6).

The role of the gut microbiome in SCD pathophysiology and its interplay with inflammation is an area of ongoing investigation. In mouse studies, our group previously demonstrated that gut microbial antigens play a key role in increasing the number of AN (7). Depletion of gut microbes using broad-spectrum antibiotics decreased the AN population and not only reduced vaso-occlusion but also decreased organ damage and sequelae of sepsis.

Building on this background, we compared differences in cytokines, chemokines, adhesions molecules, aged neutrophils and community level metrics of the gut microbiome in SCD patients treated with HU vs CT and their impact on markers of disease. We hypothesized that there would be differences in aged neutrophils, inflammatory cytokines and chemokines, and markers of gut microbiome diversity in patients treated with HU versus those on CT.

## Methods

This is a sub study of a larger cohort of SCD patients. The study was approved by the Einstein Montefiore Institutional Review Board (IRB# 2018-9080) and parents or patients provided written informed consent and assent as age appropriate. Pediatric patients with SCD (severe genotypes SS and Sβ0 thalassemia) aged from 8 to 21 years old, with no acute complications (infection, VOC) who were on HU or receiving CT were included in this sub study. Those in the CT arm were on chronic transfusions for at least 6 months and had been transfused within the last 6 weeks. Typical indications for CT were cerebral vaso-occlusion, abnormal transcranial dopplers, nocturnal hypoxemia, chronic pain, or recurrent acute VOC. 14 children with SCD undergoing monthly red blood cell transfusions and 40 children on HU therapy alone were included; demographic and clinical characteristics are described in **Table 1**. None of the patients in the CT cohort were on HU therapy at the time of study enrollment and sample collection. Participants provided blood and stool samples, the latter within two weeks from the blood sample collection. Patients were not on antibiotics in the preceding 2 months prior to sample collection.

**Table 1:** Baseline characteristics of patients by treatment group.

	Total	Hydroxyurea group	Chronic transfusion group
Total number	54	40	14
Age (years)	13.21±4.46	12.69±4.54	14.67±4.04
Body mass index	18.58±3.94	18.26±3.87	18.98±4.09
Number of males	27	20 (50%)	7 (50%)
Race	American Native	1 (2.5%)	0 (0%)
	White	0 (0%)	0 (0%)
	Black	29 (72.5%)	13 (93%)
	Other/Unknown	10 (25%)	1 (7%)
HbSS Genotype	53	39 (97.5%)	14 (100%)

### Quantification of immune mediators and soluble adhesion molecules

Venous blood in Acid Citrate Dextrose was collected from study participants. Plasma concentration (pg/ml) of G-CSF, IFNγ, IL-10, IL-17α, IL-1α, IL-1β, IL-6, IL-8, IP-10, MCP-1, MIP-1β, TNFα were measured using MILLIPLEX Human Cytokine/Chemokine/Growth Factor Panel A kit (MilliporeSigma, cat # HCYTA-60K). Samples were processed and run according to the manufacturer’s guidelines. Data were acquired on a Luminex Magpix Multiplex Reader (Luminex Technologies) and analyzed using Belysa Immunoassay Curve Fitting Software using a 5-parameter logistic curve fitting method (Millipore Sigma). Interpolated concentrations of all analytes were log transformed before analysis to reduce non-normal skew.

Soluble V-CAM-1, P-selectin and E-selectin concentrations were measured in plasma using commercial ELISA following manufacturer’s guidelines (Invitrogen, cat #: KHT0601 for soluble V-CAM-1, cat #: BMS219–4 for soluble P-selectin and cat #: BMS205 for soluble E-selectin).

### Aged Neutrophil number and percentage assessment

Total numbers of neutrophils and aged neutrophils were quantified in blood by flow cytometry using a LSRII Flow Cytometer equipped with FACS Diva 8.0.1 software (BD Biosciences) and analyzed with FlowJo software (Tree Star). Neutrophils were identified by forward and side scatter characteristics combined with CD16b expression, and the aged neutrophil subset evaluated by CD62Llo CXCR4hi expression within the neutrophil population as published previously (7). Whole blood from the same sample used for flow cytometry assays was diluted 1:10 in PBS for complete blood count with differential counts on ADIVA 120 (Siemens Healthcare Diagnostics).

### Metagenome profiling

Stool samples were processed and sequenced on the Illumina HiSeq platform (2 × 150 bp paired-end reads) by the Molecular Microbiology Facility of the Integrated Genomics Operation at Memorial Sloan Kettering Cancer Center. Taxonomic profiling of bacterial communities was conducted with the bioBakery 3 suite (8). Raw reads were quality-filtered and screened for human contamination using KneadData (v0.10.0) with -t 8 -p 12 –cat-final-output. Taxa were then profiled with MetaPhlAn3 (v3.0) using –no_map –nproc 12 and the mpa_v30_CHOCOPhlAn_201901 clade-specific marker database. MetaPhlAn outputs were merged into a single table using merge_metaphlan_tables.py. Shannon diversity index (SDI) was calculated with calculate_diversity.R script with parameters -d alpha -m shannon., and the Firmicutes: Bacteroidetes (F:B) ratio was computed by dividing the relative abundance of ‘Firmicutes’ by that of ‘Bacteroidetes’.

### Statistical data analyses

We used Student t-test and/Wilcoxon rank-sum test for normally and not normally distributed variables, respectively, to compare means and medians/distributions between the two groups of patients receiving HU vs CT. Chi-square tests were used to compare proportions for categorial variables between the two groups. All tests were 2-sided using p<0.05 as statistically significant. Spearman correlations were calculated using GraphPad Prism (V10.5.0, GraphPad Software, L Jolla, CA). Median regression analysis was conducted to examine the relationship of aged neutrophil numbers with neutrophil numbers, ferritin, percent transferrin saturation and treatment arm. This model relaxes the normal assumptions of the outcome (i.e., aged neutrophil number). Variables with p<0.05 in bivariate analyses were entered in the multivariate model; the final model included only variables that had a p<0.05. Multiple linear regression models were also built to identify mediators (cytokines, chemokines, and SDI) associated with total number of neutrophils and total number of AN. The final model included only variables that had a p<0.05. Models were done using STATA Software, version 15.1.

## Results

### Study cohort, clinical laboratory findings

There were 54 SCD patients from the larger parent study that met inclusion criteria for this sub analysis comparing HU (n=40) and CT (n=14) (**Table 1**). The two groups were similar in age, race, sex and BMI. One patient in the HU group had HbSβ0; all others were genotype HbSS. Five patients were on CT for secondary stroke prophylaxis and 3 for primary stroke prophylaxis (history of abnormal TCD and severe CNS vasculopathy). Four patients were on CT for recurrent acute pain episodes and 2 for nocturnal hypoxemia with associated neurocognitive dysfunction. Twelve of the CT patients had prior exposure to HU therapy and 2 patients with history of stroke had never received HU. Only one of these 12 patients had discontinued HU therapy previously secondary to complaints of nausea and dizziness.

Clinical laboratory data is shown in **Table 2**. As expected, there was a statistically significantly higher HbF% in the HU group and a lower HbS% in the CT group. In addition, hemoglobin, ferritin and transferrin saturation were significantly higher in the CT group. There was also a statistically significantly higher white blood cell count and absolute reticulocyte count in the CT cohort (**Table 2**). There were no statistically significant differences in platelet number, total or direct bilirubin, or lactate dehydrogenase (LDH) between the two groups.

**Table 2:** Clinical laboratory results for study participants, median (interquartile range).

	Hydroxyurea N=40	Chronic transfusions N=14	P- value
Lactate dehydrogenase (unit/L)	499 (384.5-632.5)	429 (360-483)	0.099
Platelet (×10^6^/µL)	382 (317.5-468.5)	424.5 (369-505)	0.18
Hemoglobin (g/dL)	8.7 (7.95-9.35)	9.4 (8.7-9.9)	**0.035**
HbS (%)	79 (77.35-82.5)	37.6 (27.4-37.6)	**<0.0001**
HbF (%)	14.05 (11.8-16.45)	4.6 (3.5-6.5)	**<0.0001**
WBC (x10^3^/µL)	5.9 (4.8-7.8)	9.8 (8.1-11)	**0.0005**
Absolute Reticulocyte (x10^3^/µL)	261.5 (184.4-312.9)	370.5 (220.8-431.9)	**0.008**
Total Bilirubin (mmol/l)	2.2 (1.8-3.7)	2.7 (0.05-3.95)	0.054
Indirect Bilirubin (mmol/l)	1.8 (1.03-3.4)	2.4 (1.6-3.3)	0.078
Ferritin (ng/mL)	109.5 (109-294)	1850 (1325-3966)	**<0.0001**
Transferrin saturation (%)	34 (23-50)	73 (50-91)	**0.0001**
Total neutrophil count (x10^3^/µL)	2443 (1779-3895)	5275 (3928.5-6649)	**0.0004**
Aged neutrophils (x10^3^/µL)^1^	465 (190-845)	2823 (2013-3996)	**<0.0001**
Aged neutrophils as percentage of total neutrophils^1^	16% (8-16)	57% (52-79)	**<0.0001**

^1^One hydroxyurea-treated patient did not have data on aged neutrophils.Bolded values are p < 0.05 and statistically significant.

Consistent with our primary hypothesis, the total neutrophil count, AN and AN as a percentage of the total neutrophil account were significantly lower in the HU cohort compared to the CT group (p=0.0004, p < 0.0001 and p < 0.0001, respectively, **Table 2**).

### Comparison of concentrations of soluble adhesion molecules, cytokines and chemokines in HU compared to CT treated SCD patients

**Figure 1:**
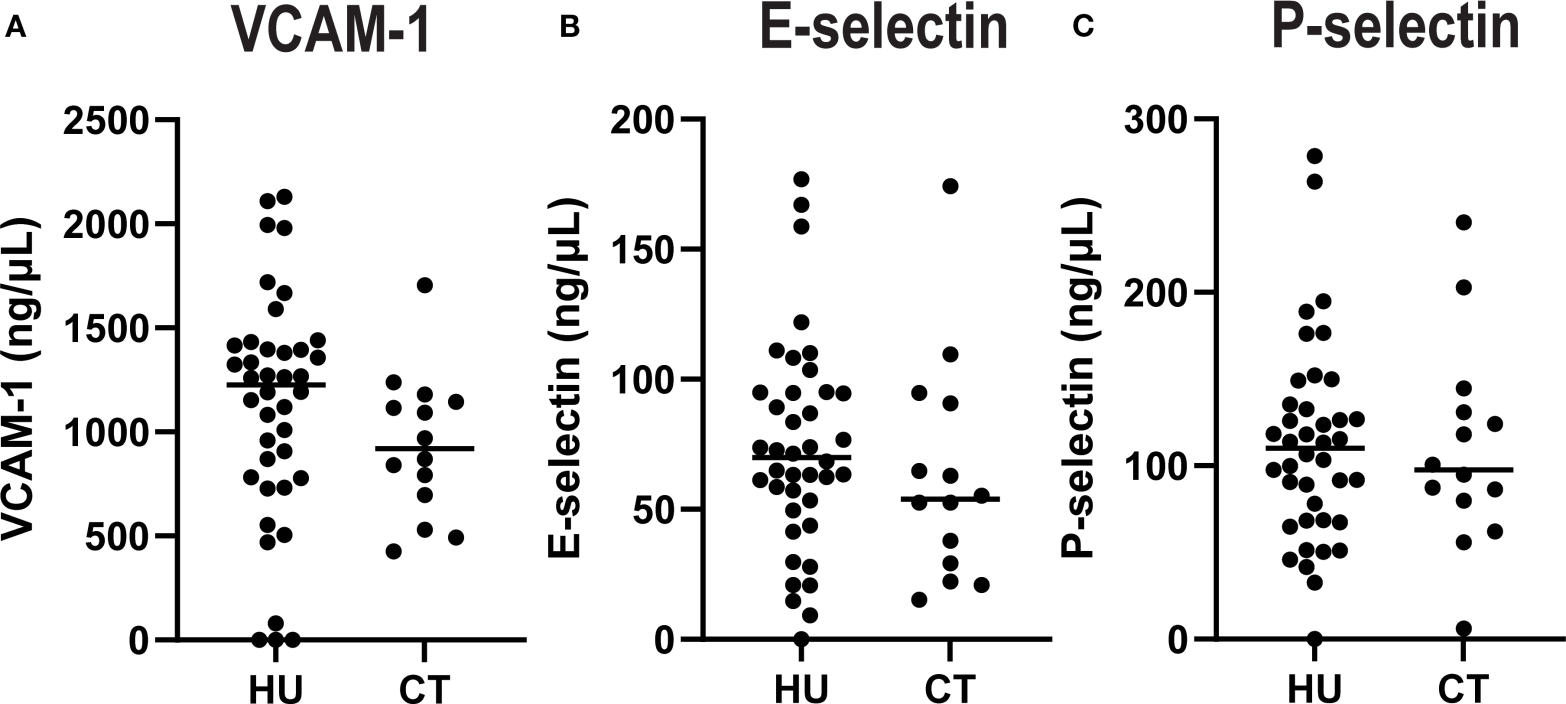
Plasma concentrations of adhesion molecules by treatment group. Soluble VCAM **(A)**, E-Selectin **(B)**, and P-Selectin **(C)** were quantified in plasma. Each dot indicates an individual patient, and the line indicates the median for each group.

**Figure 2:**
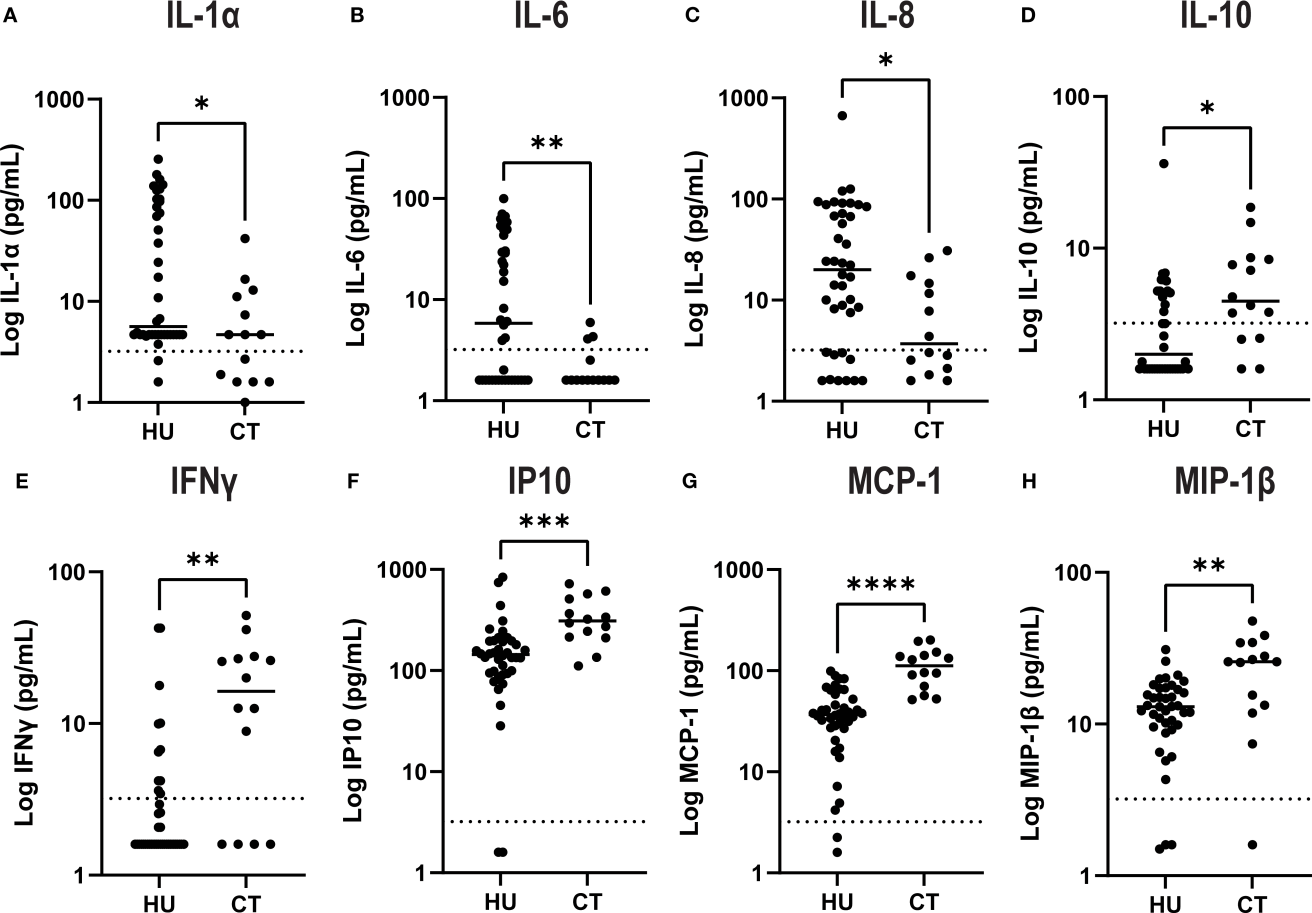
Plasma levels of select cytokines and chemokines by treatment group. Cytokines **(A–E)** and chemokines **(F–H)** were quantified in plasma by Luminex assay. Results for each individual patient are shown in the scatter plot and the line shows the median for each group. The asterisk indicate significance by unpaired-t-test (*p<0.05, **p< 0.01, *** p< 0.001 and ****p< 0.0001).

There were no statistically significant differences in plasma concentrations of soluble adhesion molecules comparing HU vs CT patients (**Figure 1**). However, there were significant differences in levels of several cytokines and chemokines (**Figure 2**). HU patients had significantly higher levels of the cytokines IL-1 α, IL-6 and the chemokine IL-8 (CXCL8), which are typically associated with inflammation, but lower levels of the other chemokines, IP-10 (CXCL10), MCP-1 (CCL2), and MIP1β (CCL4) as well as lower levels of IFN-γ and IL-10. More than half of the patients in both groups had undetectable levels of TNF, GCSF, IL-17 and IL-1β. and thus these were not further analyzed. Either the levels were low in the subjects at baseline or this represents a salutary effect of both treatments as Dembele et al. saw significantly elevated levels of TNF and IL-1β levels in SCD patients on transfusions and no treatment compared non-SCD controls (4).

### Markers of gut diversity do not differ in SCD treated with HU compared to CT

**Figure 3:**
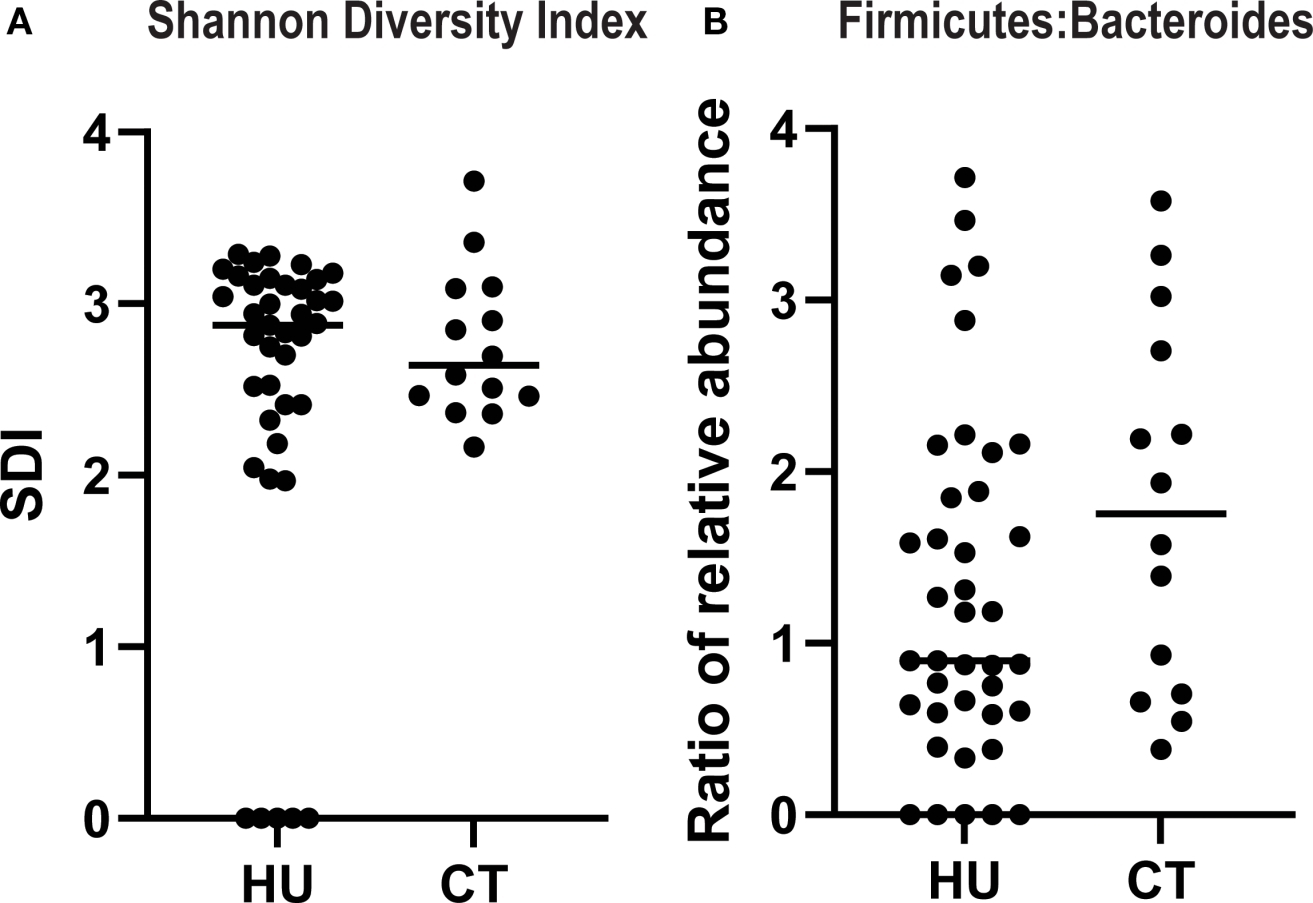
Alpha diversity and ratio of firmicutes to bacteroides in SCD patients treated with hydroxyurea versus chronic transfusions. The Shannon diversity index **(A)** and relative abundance of firmicutes and bacteroides species **(B)** were quantified from the 16sRNA sequencing data. Results for each individual patient are shown and the line represents the median.

A less diverse microbiome and imbalance in the ratio of Firmicutes: Bacteroides (F:B) are markers of gut microbial dysbiosis sand have been linked to inflammatory states (9). Thus, we focused on those two measures to assess if there were any association between treatment and these markers There were no statistically significant differences in the SDI or ratio of F:B in the two treatment groups, although there was a nonsignificant trend towards higher F:B ratio in patients treated with CT (p=0.13, Mann-Whitney) (**Figure 3**).

### Correlation analyses

**Figure 4:**
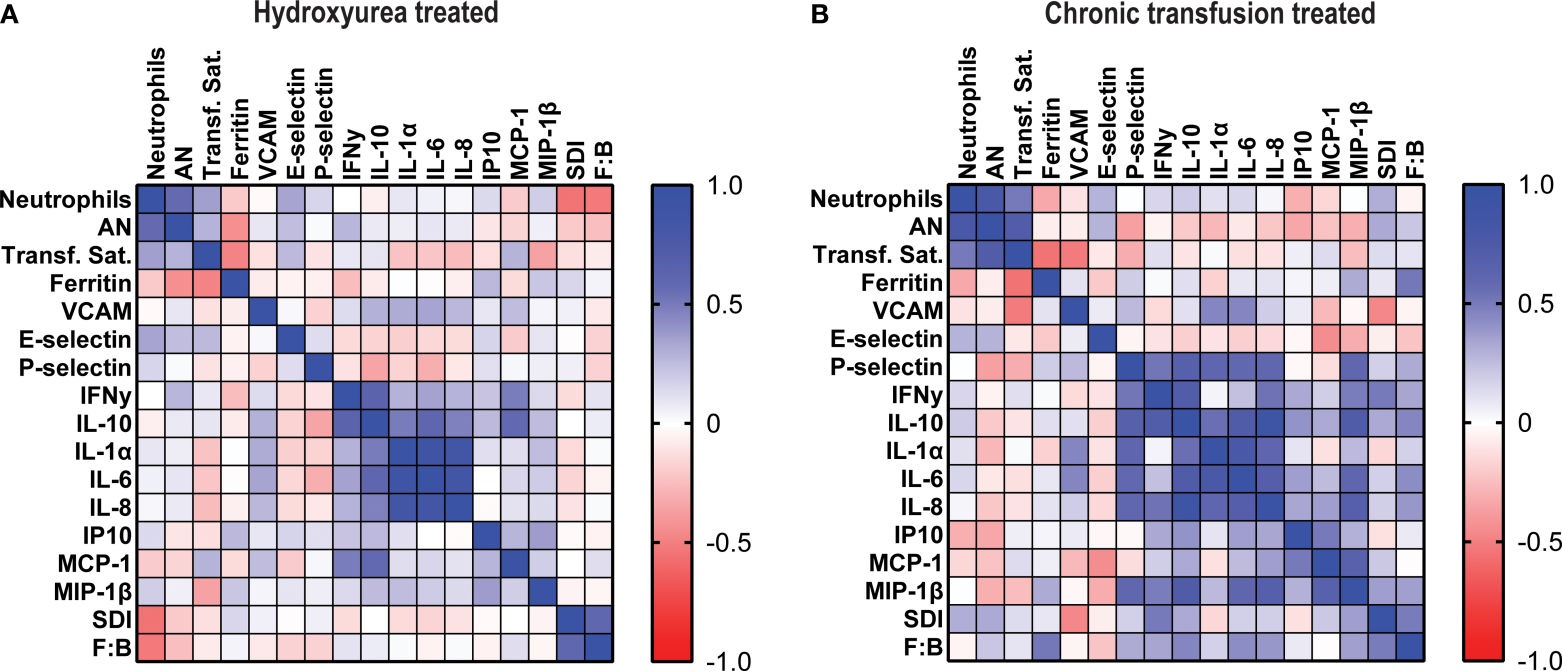
Total neutrophils correlate negatively with Shannon diversity index and Firmicutes: Bacteroides ratio in hydroxyurea treated cohort. Heatmaps showing Spearman correlation coefficients between total and aged neutrophils, transferrin saturation, ferritin, immune mediators, Shannon diversity index and F:B ratio in **(A)** hydroxyurea treated and **(B)** chronic transfusion treated patients.

Among the patients treated with HU, Spearman correlation coefficients showed that the total neutrophil count was associated positively with AN (p< 0.0001) and negatively with SDI (p< 0.0001) and F:B ratio (p< 0.001) but not with any of the cytokines or chemokines. In contrast, AN only correlated with total neutrophil count. There were strong correlations between several cytokines and chemokines. Among the patients treated with CT, the neutrophil count and AN correlated with one another but not with any of the mediators or with SDI or F:B. (**Figure 4**).

### Bivariate and multivariate regression models

Combining all patients (HU and CT), there were statistically significant positive relationships between AN and total neutrophil numbers (β = 0.472, p < 0.0001) as well as ferritin (β = 0.531, p = 0.001), but not with percent transferrin saturation (p = 0.28). These associations were interrogated to evaluate the independent association of aged neutrophils with treatment cohort using relevant clinically available variables. Hydroxyurea reduces the total neutrophil count and iron overload from transfusions is a potential confounder as it can drive inflammation. We initially added ferritin into the multivariate model, but due to the large p-value, we removed it. In the final multivariate median regression analyses, there was a strong statistically significant inverse association between aged neutrophil and HU vs CT group (β = -1485.76, p < 0.0001) after adjusting for total neutrophil numbers (β = 0.322, p = 0.001). Neither ferritin nor percent transferrin saturation were associated with AN and therefore were not included in the final models.

We also explored the association between treatment arm, cytokines, chemokines and SDI with AN and total neutrophils in a multivariable linear regression model. As shown in **Table 3**, being on CT was associated with a significant increase in both AN and total neutrophil count. In addition, the SDI was negatively associated with total neutrophils in both the initial and final model (after removing nonsignificant variables).

**Table 3:** Variables associated with aged neutrophils and total neutrophil count.

Initial model				
Dependent variable	Aged neutrophils		Total neutrophils	
Model R^2^ (p-value)	0.55 (0.0001)		0.47 (0.002)	
	β-coefficient	P-value	β-coefficient	P-value
Treatment group (0= HU, 1=CT)	2914±596	<0.0001	3089±1019	0.004
IL-1α	-1.45±11	0.9	4.89±18.7	0.8
IL-6	6.5±31	0.84	-27±52	0.6
IL-8	0.45±2.5	0.86	4.0±4.1	0.34
IFN-γ	16±23	0.47	19.3±38	0.62
IL-10	-14±46	0.77	44.9±77.2	0.57
IP-10	-0.91±1.2	0.46	-0.96±2.0	0.64
MCP-1	-2.7±6	0.66	-3.2±10.2	0.76
MIP-1β	-16.7±23	0.47	-5.6±38.5	0.88
Shannon diversity index	18±189	0.93	-1335.7±319.6	<0.0001
Constant	980±626.5	0.125	6620.6±1954.6	<0.0001
Final model				
Dependent variable	Aged neutrophils		Total neutrophils	
Model R^2^ (p-value)	0.51 (< 0.0001)		0.44 (<0.0001)	
	β-coefficient	p-value	β-coefficient	p-value
Treatment group	2500±352	<0.0001	3020±607	<0.0001
Shannon diversity index	14.8±167	0.93	-1214.5±279.8	<0.0001
Constant	613±447.5	0.18	6105.5±748.6	<0.0001

## Discussion

In this study, we compared select markers of inflammation include total and ANs in SCD patients treated with HU versus those receiving monthly transfusion therapy. Prior research has established that HU has anti-inflammatory properties, primarily through the reduction of leukocyte counts and by limiting the expression and activity of adhesion molecules on red blood cells (RBCs), leukocytes, and endothelial cells (10). Additionally, HU has been shown to decrease levels of inflammatory molecules such as endothelin-1, TNF-α, IL-1β, IL-17, and GM-CSF, which are all critical mediators in the inflammatory cascade associated with SCD (11–13). While CT has been shown to improve inflammation in several studies, authors did not perform a direct comparison to a HU cohort. For instance, Lee et al. reported on an improvement in neutrophil degranulation in a cohort of adult SCD patients undergoing exchange transfusions when compared to pretransfusion levels (14). Hyacinth et al. examined soluble markers of endothelial activation in a cohort of 40 children on CT and compared them to 40 children on standard care. They saw improvement in inflammatory profiles in the CT cohort when compared to children receiving standard care, however details about hydroxyurea use are not presented (15).

We found that chronically transfused patients exhibited significantly higher total neutrophils and total and percentages of AN compared to those on HU, even after adjusting for total neutrophil counts, ferritin, a broad panel of cytokines, chemokines, and microbial diversity metrics. These findings suggest that chronic transfusion may be associated with a heightened neutrophil-driven inflammatory profile.

Aged neutrophils represent a senescent subset with increased integrin activation and a higher propensity for forming neutrophil extracellular traps (NETs), both of which have been implicated in the pathogenesis of inflammatory diseases (7). Their enrichment in transfused patients raises important questions about the long-term immunologic consequences of transfusion-based therapy. While hydroxyurea is known to modulate leukocyte activation and reduce inflammatory complications, our findings suggest that transfusion does not confer the same anti-inflammatory benefit at the neutrophil level.

We also observed significant elevation of several inflammatory chemokines that have been previously linked to SCD pathophysiology, including IP-10, MIP-1β, and MCP1. The inflammatory cytokine IFN-γ was also higher in the transfusion cohort. MCP1, in particular, plays a key role in recruiting leukocytes to the vascular endothelium, exacerbating vascular inflammation and promoting vaso-occlusion (16–18). In our cohort, several but not all cytokines/chemokines detected in the plasma were consistently higher in transfused patients, paralleling the increased burden of aged neutrophils and total neutrophil counts. Specifically, the pro-inflammatory cytokines, including IL-6, IL-8, and IL-1α, were higher in HU patients, while IL-10, an anti-inflammatory cytokine, was reduced, suggesting an incomplete anti-inflammatory effect of HU treatment. Variable adherence to hydroxyurea therapy may be a contributing factor that in the real world is often a consideration in escalating to chronic transfusion therapy.

Our findings regarding the gut microbiome provide additional insight into the inflammatory milieu in SCD. We observed that SDI and F:B ratio were both negatively associated with total neutrophil count in the HU group (SCC), and in the multivariate model there was a significant decrease in the total neutrophils as the SDI increased, suggesting that the loss of diversity may contribute to systemic neutrophil-driven inflammation. This is consistent with prior studies linking low alpha diversity to heightened immune activation and chronic inflammatory states (19). The F:B ratio represents the balance between two dominant bacterial phyla and has been implicated in various health and disease contexts such as obesity, inflammatory bowel disease and SCD (9, 20, 21). These findings reinforce the potential value of interventions aimed at preserving or restoring microbial diversity and composition as a strategy to modulate inflammation and improve clinical outcomes in this population. These observations need to be confirmed in larger cohorts.

There are several limitations to this cross-sectional study, the small number of patients on CT and that the patients on CT for stroke prophylaxis could have a different inflammatory profile *a priori* or more severe disease. In addition, we selected a relatively small panel of immune mediators. A prospective longitudinal study that also incorporates an unbiased plasma proteomic analysis may provide deeper understanding of how different treatments contribute to reduction of inflammation and risks for vaso-occlusive events. However, despite these acknowledged limitations, based on the results of this study we hypothesize that combining HU with transfusion could potentially mitigate inflammation. We suggest that this approach deserves further evaluation in future prospective studies. Finally, the association of aged neutrophils, an independent marker of inflammation, with the total neutrophil count provides further rationale for titrating HU dose to maximum tolerate dose to minimize inflammation.

## Data Availability

The raw data supporting the conclusions of this article will be made available by the authors, without undue reservation.
